# Supplementary material for: Integrated analysis of the aqueous humor microbiome and lens capsule transcriptome in high myopia cataract: a pilot study
Source: Front Med (Lausanne). 2026 Jun 16;13:1845205. doi: 10.3389/fmed.2026.1845205 (PMC13314463; doi:10.3389/fmed.2026.1845205)
Supplement: Supplementary file 3 [file Data_Sheet_1.zip › 6.PICRUSt2/Meta_Statistic/C372089/Wilcoxon/KO_abundance.Wilcoxon.pdf]

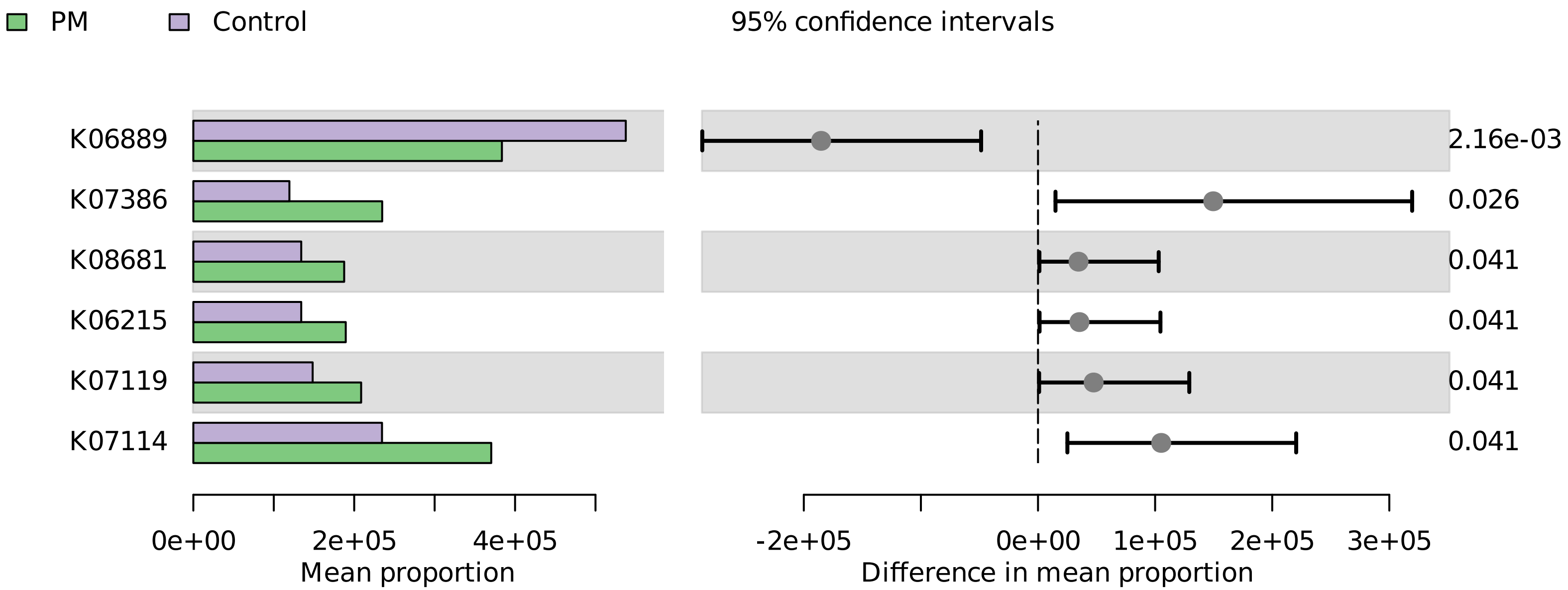

PM
Control
95% confidence intervals
K06889
K07386
K08681
K06215
K07119
K07114
2.16e-03
0.026
0.041
0.041
0.041
0.041
0e+00
2e+05
4e+05
Mean proportion
-2e+05
0e+00
1e+05
2e+05
3e+05
Difference in mean proportion
